# Quantitative Volumetric Assessment of Ablative Margins in Hepatocellular Carcinoma: Predicting Local Tumor Progression Using Nonrigid Registration Software

**DOI:** 10.1155/2019/4049287

**Published:** 2019-09-19

**Authors:** P. Hendriks, W. A. Noortman, T. R. Baetens, A. R. van Erkel, C. S. P. van Rijswijk, R. W. van der Meer, M. J. Coenraad, L. F. de Geus-Oei, C. H. Slump, M. C. Burgmans

**Affiliations:** ^1^Department of Radiology, Leiden University Medical Center, Leiden, Netherlands; ^2^Technical Medicine, University of Twente, Enschede, Netherlands; ^3^Department of Gastroenterology and Hepatology, Leiden University Medical Center, Leiden, Netherlands; ^4^Biomedical Photonic Imaging Group, TechMed Centre, University of Twente, Enschede, Netherlands; ^5^Department of Robotics and Mechatronics, University of Twente, Enschede, Netherlands

## Abstract

**Purpose:**

After radiofrequency ablation (RFA) of hepatocellular carcinoma (HCC), pre- and postinterventional contrast-enhanced CT (CECT) images are usually qualitatively interpreted to determine technical success, by eyeballing. The objective of this study was to evaluate the feasibility of quantitative assessment, using a nonrigid CT-CT coregistration algorithm.

**Materials and Methods:**

25 patients treated with RFA for HCC between 2009 and 2014 were retrospectively included. Semiautomated coregistration of pre- and posttreatment CECT was performed independently by two radiologists. In scans with a reliable registration, the tumor and ablation area were delineated to identify the side and size of narrowest RFA margin. In addition, qualitative assessment was performed independently by two other radiologists to determine technical success and the anatomical side and size of narrowest margin. Interobserver agreement rates were determined for both methods, and the outcomes were compared with occurrence of local tumor progression (LTP).

**Results:**

CT-CT coregistration was technically feasible in 18/25 patients with almost perfect interobserver agreement for quantitative analysis (*κ* = 0.88). The interobserver agreement for qualitative RFA margin analysis was *κ* = 0.64. Using quantitative assessment, negative ablative margins were found in 12/18 patients, with LTP occurring in 8 of these patients. In the remaining 6 patients, quantitative analysis demonstrated complete tumor ablation and no LTP occurred.

**Conclusion:**

Feasibility of quantitative RFA margin assessment using nonrigid coregistration of pre- and postablation CT is limited, but appears to be a valuable tool in predicting LTP in HCC patients (*p*=0.013).

## 1. Introduction

Radiofrequency ablation (RFA) has been recognized as first line treatment for very early-stage hepatocellular carcinoma (HCC) (lesion diameter <2 cm) and is used as treatment for unresectable early-stage HCC (solitary lesion, or a maximum of 3 lesions with a diameter ≤3 cm each), according to the Barcelona Clinic for Liver Cancer (BCLC) staging system [1, 2]. As a result of the implementation of surveillance in high-risk populations, diagnosis of BCLC very early- or early-stage HCC is now feasible in up to 60% of all new HCC cases in developed countries [3]. This makes RFA an increasingly used treatment modality. Recurrence rates for RFA in very early-stage HCC patients are comparable to those after surgical treatment [1]. However, higher recurrence rates are found in patients treated for larger HCC lesions [4–6].

After RFA treatment, two types of intrahepatic recurrences may occur. Local tumor progression (LTP) is found in up to 50% of ablations [7] and is known to be associated with insufficient ablative margin, large tumor size, blood vessels in the direct proximity of the tumor, and adhesion of viable tumor cells to the RFA electrodes [8]. Distant intrahepatic recurrence is related more to systemic parameters, such as the presence of vascular invasion, multifocal disease, elevated alpha-fetoprotein blood levels, and hepatitis C viral infection [9].

The preferred treatment for early-stage HCC is surgical resection. However, many patients are not eligible for this treatment, due to cirrhosis with portal hypertension, unfavorable tumor location, and/or comorbidities [1, 10]. Thermal ablation is considered as the treatment of choice for unresectable early-stage HCC up to 5 cm. Distant intrahepatic recurrence rates after resection and ablation are similar, but LTP rates are higher after ablation and negatively affect overall survival [4–6, 11]. To improve the results of RFA in unresectable early-stage HCC, a reduction of LTP rates appears to be crucial.

Histological confirmation of total tumor necrosis after RFA is not possible. In many centers, the current workflow involves qualitative assessment of RFA margins by scrolling through pre- and postinterventional images, separately. Technical success is considered when a predefined amount of energy is successfully delivered to the tumor, and complete tumor coverage with sufficient ablative margins is confirmed on contrast-enhanced computed tomography (CECT) [8]. In general, an ablative margin of >5 mm, or ideally 10 mm, is recommended [8]. These values are rather arbitrarily derived from surgical standards and supported by some studies [10–12]. However, the evidence is limited, and no standardized way of ablative margin assessment is currently available.

Supportive ablation verification software has gained interest. However, at this moment, software dedicated to quantitative ablation margin assessment is lacking and available software has not been validated in large patient cohorts. Merging of pre- and postablation scans can be performed using either nonrigid or rigid coregistration software. Nonrigid coregistration algorithms allow more degrees of freedom in the transformation to fit a scan better onto another. Besides global linear transformations, like translation and rotation, the algorithm may, e.g., use radial basis functions or other free form deformation models that allow for local warping of the image to find a better registration. Mirada RTx (Mirada Medical Ltd., Oxford, UK) is a software application developed for radiation therapy treatment planning that uses nonrigid registration of medical image datasets including computed tomography (CT) and magnetic resonance imaging (MRI). This software was used in this study.

The primary objective of this study was to assess the feasibility of quantitative three-dimensional (3D) margin assessment after nonrigid CT-CT coregistration of pre- and postinterventional imaging, using Mirada RTx. Secondary objectives were to compare quantitative ablative margin assessment with the current workflow of qualitative assessment and to assess whether quantitative assessment allows prediction of local tumor progression.

## 2. Methodology

### 2.1. Patients

All patients that were consecutively treated with RFA for de novo HCC between January 2009 and March 2014 (*n* = 79) in our institution were identified retrospectively. The diagnosis of HCC was based on either histology or radiological findings according to European Association for the Study of the Liver (EASL) criteria (arterial enhancing lesion >1 cm with washout on the late phase on CT or MRI). Exclusion criteria were multifocal disease (*n* = 27), surgical approach (*n* = 4), adjuvant trans-arterial chemoembolization (TACE) (*n* = 7), lateral patient positioning on the postablation scan (*n* = 11), and extensive metal artifacts caused by in-vivo RFA probes (*n* = 5). Finally, 25 patients were included in this study. Baseline characteristics of this cohort are shown in Table 1. Pre- and postablation multiphase CECT scans with an arterial and portal venous phase were available for all patients.

### 2.2. RFA Procedure

Percutaneous RFA procedures were performed under general anesthesia and with image guidance of ultrasound and/or CT. Based on tumor size and availability, one of the single electrode RFA systems (3 cm exposed tip Cooltip (Covidien Ltd., Gosport, Hampshire, United Kingdom)) or StarBurst XL (AngioDynamics, Amsterdam, Netherlands)) or multiple electrode RFA systems (3 or 4 cm exposed tip Cooltip with switch control system (Covidien Ltd.)) was used. The ablation time was set 12 minutes for single Cooltip electrode and 16 minutes for the multiple Cooltip electrodes. Temperature-based ablation was performed with the StarBurst XL electrode.

Immediately after ablation, a CECT scan of the liver was performed on a 16-slice spiral CT (Aquilion-16, Toshiba, Tokyo, Japan) with the following settings: 120 kV, rotation 0.5 s, and 16 × 1 mm scanning. Dose weight-dependent Ultravist 370 contrast agent or Xenetix 350 contrast agent was used with a 15-second and 75-second delay after bolus triggering for arterial phase and portal venous phase, respectively. Consequently, the CECT scans were qualitatively evaluated for technical success. The ablation was considered technically successful if the coagulation area fully encompassed the tumor in the absence of residual tumor enhancement. This assessment was done by visual comparison of the tumor location on preprocedural CT and area of necrosis on the postprocedural CT (“eyeballing”) and 2D measurements.

### 2.3. Follow-Up

All patients underwent blood tests (including alpha-fetoprotein) and CECT every three months after treatment. Upon discretion of the referring physician or interventional radiologist, multiphase MRI was used instead of CECT. Liver explants of patients that underwent an orthotopic liver transplantation (OLTx) were pathologically examined for local tumor progression. The median follow-up time was 9.5 months.

### 2.4. Scoring

CT-CT registration and delineation of the tumor volume and RFA ablation volume were performed in Mirada RTx software. Two radiologists independently performed the CT-CT coregistration and delineation of the tumor and RFA ablation volume, while being blinded for follow-up information. CT-CT coregistration was performed using a semiautomated nonrigid registration. Manual alterations were possible by rotation and translation of a scan or with use of a rigid landmark algorithm. The registration performance was graded on a 5-point scale (1 = completely unreliable coregistration; 2 = suboptimal coregistration; 3 = sufficient quality of coregistration, but not accurate enough for measurements in mm; 4 = good coregistration; 5 = perfect coregistration). Patients with coregistration performances of 1–3 were excluded from further analysis.

A greyscale-based semiautomatic delineation tool was used with manual adjustments for segmentation of the tumor and ablation volume. RFA margins were quantitatively assessed in a fused image window. The narrowest margin (in mm) as well as the anatomical location of the narrowest margin or largest tumor residue was determined. Interobserver agreement was determined for the categorical assessment of margin size (1: negative, 2: 0 to 5 mm, or 3: ≥5 mm). A “negative” margin was defined as tumor extending beyond the boundaries of the ablation zone on the overlay of pre- and postablation CT. This would not necessarily mean that the tumor was incompletely ablated. The ablation may have caused tissue shrinkage, and as a result, the ablation area may be smaller than the tumor even when the tumor was completely ablated. The side of LTP occurrence was correlated with the side of the minimal ablative margin or largest tumor residual. A comparison of patient characteristics between those with and without LTP was performed.

Two other radiologists independently repeated the qualitative assessment of the pre- and postablation scans for technical success and determined categorical ablative margins (1: negative, 2: 0 to 5 mm, or 3: ≥5 mm), while being blinded for follow-up information. Also, the anatomical side of narrowest margin was recorded. Interobserver agreement rates were determined for technical success and margin size. In both the quantitative and the qualitative assessment, a consensus reevaluation took place by the two radiologists for determining technical success for cases they initially disagreed on.

### 2.5. Statistics

Interobserver agreement was determined with use of unweighted Cohen's kappa statistics. A *κ* of 0 meant that the agreement was similar to chance, whereas a *κ* of 1 meant perfect agreement [13].

Continuous data were analyzed with the independent *t*-test and categorical data with the chi-square test. SPSS version 23.0 was used to perform the data analysis, and a significance interval of 5% was used. Boxplots were created using GraphPad Prism 5 (GraphPad Software, San Diego, California, USA).

## 3. Results

### 3.1. Patients

The coregistration quality of pre- and postablation scans was rated ≤3 in 7/25 (28.0%) patients, who were therefore excluded for further analysis.  Table 2 shows all patient and tumor characteristics of the 18 remaining cases that were technically feasible for quantitative analysis.

### 3.2. Scoring

The interobserver agreement for *quantitative* assessment with use of CT-CT coregistration and delineation was almost perfect, with a *κ* of 0.88 (SE: 0.12 and *p* < 0.01). Categorical agreement on the minimal margin size (negative, 0 to 5 mm, or ≥5 mm) was similar with a *κ* of 0.88 (SE: 0.12 and *p* < 0.01). A consensus reevaluation of one case led to agreement on technical success that the radiologists initially disagreed on.

The interobserver agreement of two radiologists who *qualitatively* assessed the ablative margins was moderate: 0.64 (SE: 0.33 and *p* < 0.01). Agreement on categorical margin assessment was very poor (negative, 0 to 5 mm, or ≥5 mm) with a *κ* of 0.24 (SE of 0.28 and *p*=0.16). Consensus was reached between the observers on technical success for two cases that they initially disagreed on, for further analysis.

### 3.3. Local Tumor Progression Rate

In 8 out of 18 patients (44.4%), LTP was found, either radiologically (5/8), or histologically after OLTx (3/8). In 1 (5.6%) patient, distant intrahepatic recurrence was found. Out of the 10 (55.6%) patients who did not develop recurrence, 3 underwent OLTx within 1 year after RFA (average 9.3 months).

Differences in patient and tumor characteristics were analyzed between patients who developed LTP (*n* = 8) and patients who did not (*n* = 10). No significant differences were found in patient and tumor characteristics between the groups.

**Figure 1:**
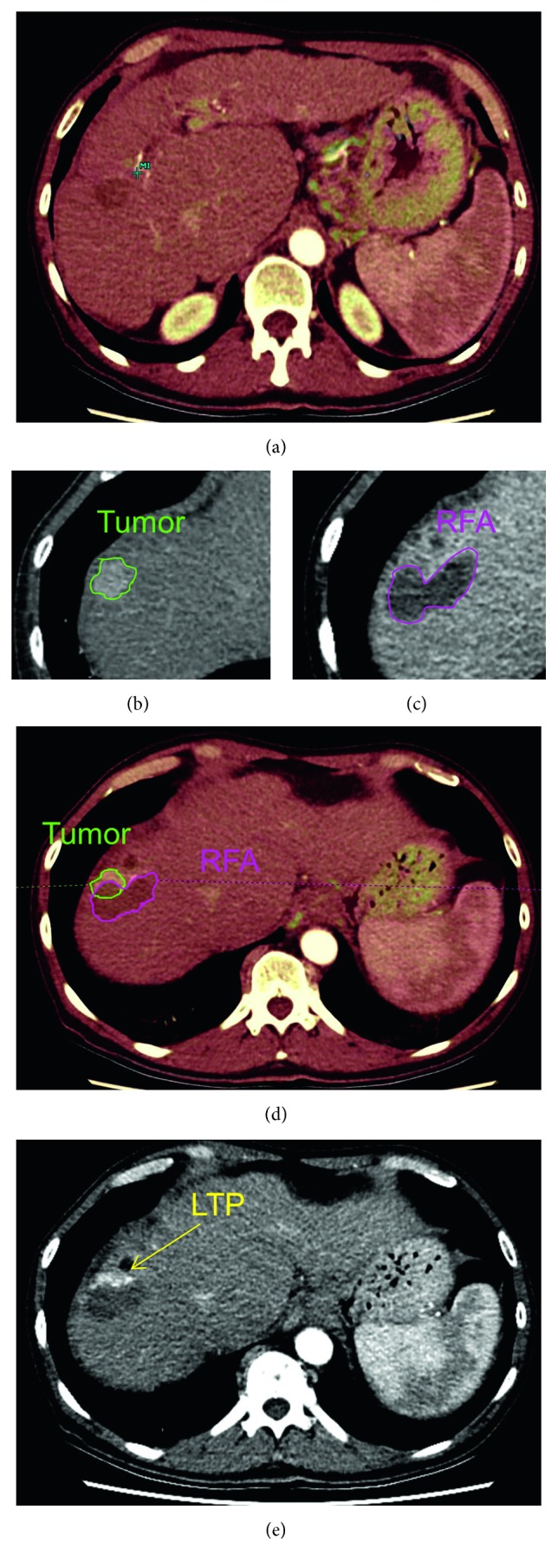
Image analysis protocol. (a) Registration (overlay) of preinterventional and postinterventional CT scans. (b) Semiautomatic delineation of tumor volume. (c) Semiautomatic delineation of RFA volume. (d) Image fusion plane: margin analysis by overlaying pre- and postinterventional imaging. (e) Follow-up scan with local tumor progression.

Based on the *quantitative* analysis, RFA necrosis fully encompassed the tumor in 6/18 (33.3%) of all patients, with a mean margin of 0.91 mm (SD: 1.11; range: 0–3 mm). In none of these patients, LTP was found. Out of the other 12 patients, 8 (66.7%) developed LTP (5 cases of LTP were identified radiologically, and 3 cases of LTP were pathologically proven after OLTx). LTP was associated with insufficient ablative margins, with a *p* value of 0.013. All patients who developed local tumor progression, did so at (one of) the anatomical side(s) with a negative ablative margin. An example of the entire workup and occurrence of local recurrence at a negative ablative margin is shown in Figure 1.

**Figure 2:**
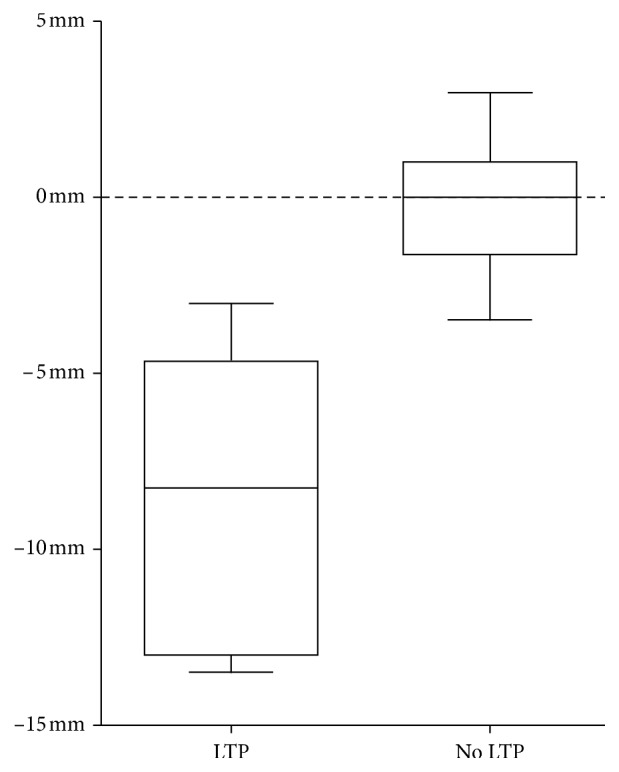
Boxplot of quantitative ablative margin size for patients with and without local tumor progression (LTP).

The average minimal ablative margin in all cases was −6.38 mm (SD: 4.64). The ablative margin size significantly correlated to the occurrence of LTP with a *p* value of 0.001. The mean ablative margin of patients who developed LTP was −8.44 mm (SD: 4.27) and −0.30 mm (SD: 2.00) for patients who did not, as can be seen in Figure 2.

Based on the qualitative analysis, 16/18 (88.9%) ablation areas fully encompassed the tumor. Yet, 6 of these patients (42.9%) developed LTP during FU. In 2 (11.1%) patients, the observers concluded that the ablation zone did not completely cover the tumor; these two patients did develop LTP.

One patient developed intrahepatic distant metastatic disease within 18 months after treatment. This was a patient with a fully ablated initial tumor with no LTP.

## 4. Discussion

In this retrospective pilot study, quantitative ablative margin assessment using Mirada RTx software was feasible only in selected patients as in 7 out of 25 patients, the performance of coregistration was insufficient. However, high interobserver agreement rates were found for quantitative assessment in the remaining 18 patients. LTP occurrence correlated with negative margin sizes with *p*=0.013, indicating a predictive value of quantitative margin assessment.

A disadvantage of minimally invasive HCC treatments is that no pathological confirmation of treatment success can be obtained. The chance on treatment success is generally thought to increase when aiming at safety margins of 5 or 10 mm, to overcome potential heat-transduction variations caused by factors such as heat sink, tumor heterogeneity, and liver parenchyma fibrosis or cirrhosis. It is challenging to accurately assess the actual ablative margins. The results of this study indicate that conventional qualitative assessment is prone to overestimation of the obtained ablative margins. Only 2 out of 8 patients who developed LTP were identified qualitatively, whereas all 8 patients were identified using quantitative assessment.

Other studies have addressed the potential of quantitative assessment of ablation margins. A rigid registration algorithm was used in the largest study, by Kim et al. [12]. They analyzed 110 HCC tumors and found a cutoff value of >3 mm as a minimal ablation safety margin. Remarkably, in only 3/110 (2.7%) ablations, the target of 5 mm safety margin was actually met. Smaller studies used a nonrigid registration algorithm similar to ours. In a retrospective study in 31 patients with HCC, nonrigid registration of pre- and postablation CT scans using Hepacare software (Siemens, Germany) was feasible with an interobserver agreement comparable to our findings [14]. In another small cohort study, correlation between margin size and LTP was evaluated in a heterogeneous cohort with different tumor types [15]. In this study, no interobserver agreement analysis was performed. To our knowledge, the current study has been the first study in which both the feasibility of using a nonrigid registration algorithm and the correlation between margin size and LTP were reviewed, in a homogeneous HCC population.

As the liver is a deformable organ, a nonrigid registration seems to be a better fit for reliable registration. The Mirada RTx software used in this pilot study is not dedicated for the quantification of ablation margins but has the tools necessary for delineation and nonrigid registration. For future research, the software should be adopted with the purpose to optimize registration of pre- and postablation scans. Adding a step for selecting the liver as volume of interest in which optimal registration should be strived for may increase the registration success for the purpose of ablation margin measurements.

In the quantitative assessment, none of the patients with a fully ablated tumor developed LTP, even in those cases where no safety margin was found. However, tissue shrinks during ablation, which influences the quantification of safety margins [16–18]. A 0 mm ablative margin on post-RFA imaging may therefore denote a fully ablated tumor with a few millimeter of margin, as a result of tissue shrinkage. To be fully able to interpret treatment success without pathological confirmation, a better understanding of heat conduction and tissue shrinkage would be necessary, as the latter seems to occur in an inhomogeneous and unpredictable way [16]. Quantification of ablative margins therefore remains arbitrary, as it may not reflect the actual distance between the boundary of the initial tumor and the boundary of the ablation area. To use the software as a decision support tool during ablation procedures, prospective studies in larger patient cohorts are needed to determine the risk of recurrence for different ablation margins and to set a standard for the optimal ablation margin.

The LTP rate of 44.4% in this study is comparable to studies with a similar patient population. In a large randomized study that included 701 patients treated with RFA, the HEAT III study, tumor progression rates of 53.3% were found after treatment with RFA in a population with slightly more unfavorable patient and tumor characteristics [19].

The main limitations of this study are its retrospective design and low sample size. Although the initial cohort consisted of 79 patients, only 25 patients were included, of which 18 patients were assessable for the final analysis. The majority of patients were excluded for this pilot study to prevent potential bias in follow-up data. Secondary exclusion (7/25 included patients) due to unfeasible registration could potentially be reduced by performing a CT scan immediately before and after the ablation. To optimize coregistration of the CT scans, the scan should be acquired with the patient in an identical position and during a similar inhalation mode or with use of high-jet ventilation.

Clinically, LTP is not the most valuable outcome measure. This study was designed as a pilot study to evaluate software that assesses the completeness of a local treatment. Therefore, LTP was chosen as the most relevant parameter for this study rather than survival.

## 5. Conclusion

Feasibility of coregistration of pre- and postablation CT images using Mirada RTx software was found for selected patients (18/25), as difference in position and shape of the liver may hamper reliable image coregistration. For patients in whom coregistration is feasible, the interobserver agreement is high, confirming the robustness of this method. Compared to qualitative assessment, quantitative assessment of ablative margins allows better prediction of LTP and may thus be a better method to determine technical success. To increase the feasibility of CT-CT coregistration as a method to determine the endpoint of ablation, there is a need for optimized scanning protocols and dedicated software prospective studies in larger patient cohorts are needed to better determine the risk of recurrence for different ablation margins and to define a cutoff value for the optimal margin.

## Figures and Tables

**Table 1:** Characteristics of analyzed patients.

	*n*	
Total	25	
Age		
Mean (SD)	62, 1	11.8
Sex		
Male	20	80.0%
Female	5	20.0%
Cirrhosis presence		
Yes	25	100.0%
No	0	0.0%
Ascites presence		
Yes	7	28.0%
No	18	72.0%
Etiology		
Hepatitis B	2	8.0%
Hepatitis C	8	32.0%
Alcohol abuse	15	60.0%
NASH	2	8.0%
Cryptogenic	1	4.0%
ECOG		
0	24	96.0%
1	1	4.0%
Child–Pugh score		
A	12	48.0%
B	13	52.0%
C	0	0.0%
BCLC		
Very early	10	40.0%
Early	15	60.0%
Lesion size (mm)		
Median (range)	20	12–45
Year of RFA		
2009–2011	10	31.3%
2012–2014	15	46.9%

NASH = nonalcoholic steatohepatitis; ECOG = Eastern Cooperative Oncology Group; BCLC = Barcelona Clinic for Liver Cancer; RFA = radiofrequency ablation. More etiological factors could be present in one patient.

**Table 2:** Characteristics of patients technically feasible for quantitative analysis.

	Total		No LTP		LTP		
	*n*		*n*		*n*		*p* value
Total	**18**		10		8		
Age							
Mean (SD)		**64.9 (9.0)**		66.1 (10.7)		63.4 (6.5)	*0.538*
Sex							
Male	**14**	**77.8%**	7	70.0%	7	87.5%	*0.375*
Female	**4**	**22.2%**	3	30.0%	1	12.5%	
Cirrhosis presence							
Yes	**18**	**100.0%**	10	100.0%	8	100.0%	
No	**0**	**0.0%**	0	No	0	0.0%	
Ascites presence							
Yes	**5**	**27.8%**	3	30.0%	2	25.0%	*0.814*
No	**13**	**72.2%**	7	70.0%	6	75.0%	
Etiology							
Hepatitis B	**0**		0		0		*0.800*
Hepatitis C	**4**		2		2		*0.410*
Alcohol abuse	**5**		2		3		*0.180*
NASH	**2**		2		0		*0.250*
ECOG							
0	**17**	**94.4%**	10	100.0%	7	87.5%	*0.250*
1	**1**	**5.6%**	0	No	1	12.5%	
Child–Pugh score							
A	**9**	**50.0%**	5	50.0%	4	50.0%	*1.000*
B	**9**	**50.0%**	5	50.0%	4	50.0%	
BCLC							
Very early	**6**	**33.3%**	3	30.0%	3	37.5%	*0.737*
Early	**12**	**66.7%**	7	70.0%	5	62.5%	
Lesion size							
Median in mm (range)		**22 (12–27)**		22 (12–27)		22 (16–25)	
OLTx <18 months							
Yes	**6**	**33.3%**	3	30.0%	3	37.5%	*0.737*
No	**12**	**66.7%**	7	70.0%	5	62.5%	
Distant intrahepatic recurrence							
Yes	**1**	**5.6%**	1	10.0%	0	0.0%	*0.357*
No	**17**	**94.4%**	9	90.0%	8	100.0%	
RFA on target quantitative assessment							
Yes	**6**	**33.3%**	6	60.0%	0	0.0%	*0.013*
No	**12**	**66.7%**	4	40.0%	8	100.0%	
RFA on target qualitative assessment							
Yes	**16**	**88.9%**	10	100.0%	6	75.0%	*0.094*
No	**2**	**11.1%**	0		2	25.0%	
Year of RFA							
2009–2011	**7**	**38.9%**	2	20.0%	5	62.5%	*0.066*
2012–2014	**11**	**61.1%**	8	80.0%	3	37.5%	

NASH = nonalcoholic steatohepatitis; ECOG = Eastern Cooperative Oncology Group; BCLC = Barcelona Clinic for Liver Cancer; RFA = radiofrequency ablation. More etiological factors could be present in one patient.

## Data Availability

The data used to support the findings of this study are included within the article.
